# The (ProteUS) Anisotropy Effect in Deep Fascia Ultrasonography: The Impact of Probe Angulation on Echogenicity and Thickness Assessments

**DOI:** 10.3390/life15050822

**Published:** 2025-05-21

**Authors:** Carmelo Pirri, Nina Pirri, Diego Guidolin, Enrico De Rose, Veronica Macchi, Andrea Porzionato, Raffaele De Caro, Carla Stecco

**Affiliations:** 1 Department of Neurosciences, Institute of Human Anatomy, University of Padua, 35121 Padova, Italy; diego.guidolin@unipd.it (D.G.); enrico.derose@studenti.unipd.it (E.D.R.); veronica.macchi@unipd.it (V.M.); andrea.porzionato@unipd.it (A.P.); rdecaro@unipd.it (R.D.C.); carla.stecco@unipd.it (C.S.); 2 Department of Medicine—DIMED, School of Radiology, Radiology Institute, University of Padua, 35121 Padova, Italy; nina_92_@hotmail.it

**Keywords:** fascia lata, deep fascia, ultrasound, imaging, anisotropy, thickness, echogenicity, tendon

## Abstract

This study investigates the influence of probe angulation on echogenicity and thickness measurements of the deep fascia, addressing methodological challenges in musculoskeletal ultrasound examination. The anisotropic nature of connective tissues can lead to distortions, affecting US imaging accuracy and diagnostic reliability. Echogenicity and thickness variations were analyzed across different probe inclinations in both transverse and longitudinal orientations. Measurements at 0° were compared with −5° and +5° angles to assess their impact on imaging consistency due to 3D-printed support. Echogenicity differed significantly with probe angulation, in particular in transverse scan at 0°, which showed substantial variation at −5° (mean diff. = 55.14, *p* < 0.0001) and +5° (mean diff. = 43.75, *p* = 0.0024). Thickness measurements also varied, reinforcing that non-perpendicular probe angulation introduces distortions. The same results were reported for longitudinal scans. These findings highlight the need for the use of standardized scanning protocols to improve reliability. The protean nature of deep fascia anisotropy, highly sensitive to minimal changes in probe orientation, necessitates precise and consistent imaging to accurately reveal its structural organization. Optimizing probe orientation is essential for advancing fascial US diagnostics.

## 1. Introduction

Ultrasound (US) examination has emerged as a fundamental imaging tool in the study of deep fascia, a highly organized connective tissue that surrounds and integrates with muscles, neurovascular structures, and skeletal components [1]. Deep fascia is characterized by densely packed, parallel-aligned collagen fibers that contribute to its mechanical strength, force transmission, and proprioceptive functions [2]. Its biomechanical function extends beyond passive support, as it actively contributes to muscle coordination and neuromuscular dynamics [2]. Given its functional significance, alterations in deep fascia are increasingly recognized as contributors to chronic musculoskeletal pain and movement restrictions, making US examination an indispensable tool for its assessment [1,2].

Deep fascia appears on US examination as a hyperechoic linear structure, typically forming a continuous interface between muscle compartments. When properly visualized, it allows for detailed examination of thickness, echogenicity and fascial mobility, enabling clinicians to identify fibrotic changes, adhesions, inflammation, and fascial stiffness. Compared to other imaging modalities, such as magnetic resonance imaging (MRI), US offers the advantage of point-of-care accessibility, affordability, and the ability to perform functional assessment, making it a preferred choice in rehabilitation and pain management. However, despite its many advantages, one of the most important technical challenges in deep fascial imaging is anisotropy.

Anisotropy is a fundamental phenomenon/artifact in musculoskeletal US examination, occurring when the reflection of US waves varies depending on the orientation of the US beam relative to the tissue’s structural alignment [3]. This effect is particularly pronounced in highly organized, collagen-rich tissues, such as tendons, ligaments, nerves, and aponeuroses, where fiber arrangement dictates the intensity and directionality of returning echoes. Unlike isotropic tissues, which exhibit uniform acoustic properties in all directions, collagenous musculoskeletal structures strongly reflect US waves only when the probe inclination angle is optimal, typically when the beam is perpendicular to the fibers. When the transducer is positioned perpendicular to a tendon or ligament, for example, the structure appears highly echogenic, with well-defined borders and a homogeneous texture. However, as the probe inclination angle deviates perpendicularly, the signal intensity progressively decreases due to increased scattering, leading to a hypoechoic or anechoic appearance. This can mimic pathological thinning, fluid accumulation or discontinuity, leading to potential misinterpretations in clinical and research settings [3]. Despite the fact that corrective measures can be performed, operator expertise remains critical in recognizing and managing anisotropic effects in musculoskeletal US examination. Awareness of which anatomical structures are prone to anisotropy and actively adjusting imaging parameters are essential to ensure accurate diagnosis and prevent misinterpretations in musculoskeletal pathology assessment [3,4,5,6,7].

The quantification of deep fascial anisotropy is relevant in conditions where fascial thickening, inflammation, or mechanical dysfunction are key diagnostic markers. The presence of anisotropic artifacts may result in false-positive or false-negative findings, leading to misdiagnosis, unnecessary interventions, or incorrect prognostic evaluations.

Given these challenges, this study aims to systematically assess the impact of anisotropy on deep fascia US examination, with a specific focus on echogenicity and thickness measurement variability across different probe inclination angles. The primary objectives of this study are (1) to quantify the degree of echogenicity variation in deep fascia as a function of probe angulation; (2) to analyze changes in measured fascial thickness at different inclination angles; and (3) to determine the optimal probe orientation for minimizing anisotropic distortions and improving the accuracy of deep fascia imaging. By addressing these objectives, this research seeks to establish objective criteria for reducing anisotropic artifacts in deep fascial US examination, thereby enhancing diagnostic accuracy and clinical applicability. Understanding how probe orientation affects the visualization of deep fascia will ultimately contribute to the development of more reliable imaging protocols, reducing diagnostic variability and improving outcomes in musculoskeletal and rehabilitative medicine.

## 2. Materials and Methods

### 2.1. Study Design

A cross-sectional study was designed in accordance with the Strengthening the Reporting of Observational Studies in Epidemiology (STROBE) guidelines [8]. The study aimed to assess and compare the echogenicity and US thickness of deep fascia of the thigh (fascial lata) of different levels (anterior region) and at different probe inclination angles (US probe) between healthy volunteers. The Helsinki Declaration and human experimentation rules [9] were considered, Ethical approval was not sought for the present study by the Ethical Committee of the Institute of Anatomy of the University of Padua because it was just an observational study without treatment and only involved volunteers. Prior to study inclusion, all participants provided written informed consent after receiving detailed information regarding the study’s objectives and US examination.

### 2.2. Study Population

Overall, 25 participants (13 female and 12 males; mean 30.45 ± 11.22 years) were recruited. Eligibility criteria mandated that participants be at least 18 years old, with the exclusion criteria being individuals who were pregnant or presented with any of the following conditions: chronic dermatological disorders (e.g., eczema, psoriasis, etc.), a history of severe lower extremity trauma, collagenopathies (including scleroderma and mixed connective tissue disorders), or chronical medical conditions necessitating ongoing pharmacological treatment. The enrollment of the subjects was performed by a specialized medical doctor with more than 8 years of experience in US imaging.

### 2.3. Ultrasound Probe Support Design and Fabrication

**Figure 1:**
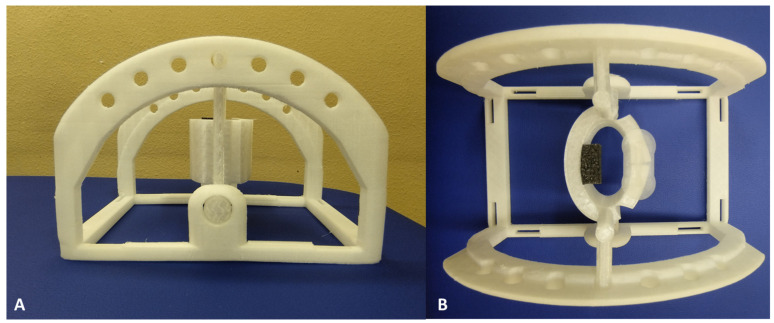
Three-dimensionally printed US probe support in PVC ((**A**) = side view; (**B**) = top view)): the support is composed of two separate parts, namely an upper section (white) specifically designed to conform to the dimensions of the US probe and change the probe inclination angles to 0°, −5°, and +5° and a lower section (white), which ensures structural stability and maintains the US probe in a suspended position relative to the surface of the skin of the healthy volunteer. All parts were designed using Autodesk Fusion 360 (Autodesk, Inc., San Rafael, CA, USA).

The support structure for US probe was designed using Autodesk Fusion 360 (Autodesk, Inc.; San Rafael, CA, USA) with the objective being to have three probe inclination angles—0°, −5°, and +5°—and to have a better interface between the probe and the surface of the volunteer. Three-dimensional computer-aided design (CAD) models were exported in STL format to facilitate 3D printing. Fabrication was performed using an Ultimaker Creality Ender 3 printer, operating at its maximum resolution setting, with polyvinyl chloride (PVC) used as the printing material. The total manufacturing process required approximately 24 h. The support consisted of two distinct components: (i) an upper section (white), specifically designed to conform to the dimensions of the US probe and change the probe inclination angles to 0°, −5°, and +5°, and (ii) a lower section (white), which ensures structural stability and maintains the US probe in a suspended position relative to the surface of the skin of the healthy volunteer (Figure 1).

A mechanical simulation of the selected material was performed using Autodesk Fusion 360 (Autodesk, Inc., San Rafael, CA, USA) to evaluate its structural response to the mechanical stresses exerted by the US probe and to ensure minimal displacement during the performance of US examination. The simulation results indicated an insignificant deformation in the component of the support, confirming its stability and structural integrity during US examination.

### 2.4. Ultrasound Examination Measurements

**Figure 2:**
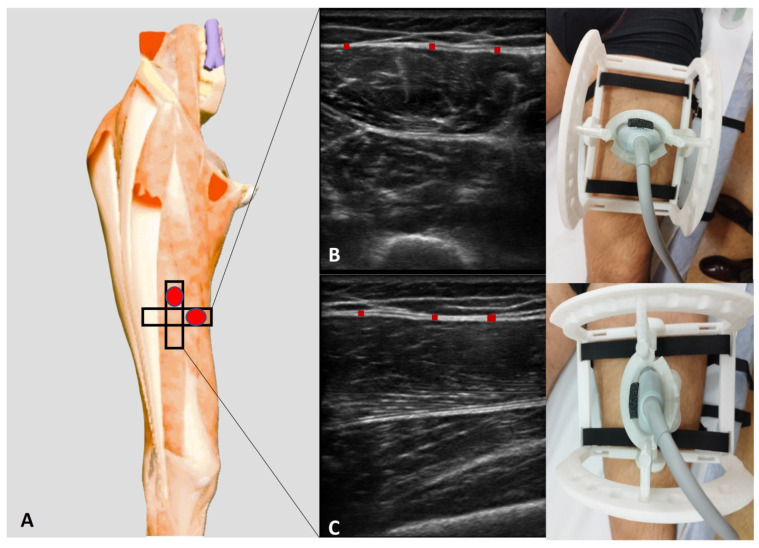
(**A**) Ultrasound protocol for performing anisotropy assessment of deep fascia. (**B**) Transversal scan (short axis). (**C**) Longitudinal scan (long axis). Red rectangle: deep fascia.

Employing an advanced high-resolution US machine (Edge II, Sonosite, FUJIFILM, Inc., Washington, WA, USA) with a frequency range of 6–15 MHz and a screen resolution of 1680 × 1050 pixels, US examination was performed at precise anatomical landmarks at the anterior region of the thigh. Furthermore, the anterior region of deep fascia of thigh was assessed following the protocol described by Pirri et al. [10]:

- Ant 2 (transversal): With the volunteer in a relaxed supine position and the right lower limb in a neutral posture, the probe was placed at the Ant 1 scanning position [10] and further moved distally along the femur. The movement followed the alignment of the rectus femoris and vastus intermedius muscles, maintaining an axial/transversal orientation. As the probe advanced, the sartorius muscle progressively disappeared from the medial aspect of the US image. At this level, the vastus medialis appeared medially, while the vastus lateralis became visible laterally, delineating the transition in muscle architecture.
- Ant 2 (longitudinal): For the longitudinal scan, the probe was rotated 90° to obtain a longitudinal view of the rectus femoris and vastus intermedius muscles. In this orientation, the rectus femoris appeared as a fusiform structure overlying the vastus intermedius, with its fibrous central tendon running parallel to the muscle fibers (Figure 2).

A specialist physician with eight years of experience in musculoskeletal and fascial ultrasonography conducted the US examinations using US probe support at the different inclination angles (0°, −5°, and +5°) in transversal and longitudinal axes. The US machine was configured in B-mode, with a propagation velocity of c = 1540 m/s, the standard for diagnostic US, and a penetration depth of 30 mm. Nylon straps were used to stabilize the US probe support on the volunteer’s surface, ensuring the acquisition of high-quality, reproducible images. To optimize acoustic coupling, a generous amount of US gel was applied.

Upon completion of each US assessment, all acquired images were frozen and systematically stored for subsequent analysis. The echogenicity and thickness of the deep fascia were quantitatively assessed using ImageJ analysis software (Version 1.54m) (available online: https://imagej.nih.gov/ij/, accessed on 5 February 2025). Each US image was segmented into three distinct regions, within which three optimally visible measurements points were identified, recorded, and averaged. To minimize the potential bias introduced by thickness variations, three equidistant points were selected within each image, and their averaged values were used for further analysis. Echogenicity was evaluated in transverse and longitudinal scans. For this purpose, the entire deep fascia structure was interactively delineated based on grayscale intensity values, where each pixel ranged from 0 (black) to 255 (white). Each pixel corresponded to a real-world spatial resolution of 0.1 mm. Following a meticulous evaluation by the ultrasonographer to identify the deep fascia, the region of interest (ROI) was segmented. The mean grayscale intensity of the entire deep fascia at different levels, as well as the ROI, was considered a quantitative estimator of echogenicity.

### 2.5. Statistical Analysis

Statistical analysis was conducted using GraphPad PRIMS 8.4.2 (GraphPad Software Inc., San Diego, CA, USA), with the significance threshold set at *p* < 0.05. Effect size estimation was conducted via G*Power 3.1 (Universität Düsseldorf: Psychologie), adhering to Cohen’s kappa interpretation, categorizing effect size as small (d = 0.20), medium (d = 0.50), and large (d = 0.80) [11]. In our pilot investigation, corroborated by previous research [10], the computed effect size for thickness was d = 1, with an α error probability of 0.05, a statistical power (1-β) of 0.95, and a requisite sample size of 20 volunteers [10]. Nonetheless, our study incorporated 25 participants.

Normality was assessed using the Kolmogorov–Smirnov test. Descriptive statistics, encompassing measures of central tendency and dispersion, were computed separately for each cohort of levels, using the mean and standard deviation (SD) for parametric data representation. Comparative assessments across probe angulations were analyzed via one-way analysis of variance (ANOVA), followed by Tukey’s post hoc test for multiple comparisons. Additionally, intra-rater reliability was quantified via a two-way, mixed-model, intra-class correlation coefficient (ICC 3, k) of type C. ICC values were interpreted as follows: poor reliability (<0.5), moderate (0.5–0.75), good (0.75–0.9), and excellent (>0.90) [12].

## 3. Results

### 3.1. Ultrasound Measurements of Deep Fascia of Thigh: Echogenicity

#### 3.1.1. Echogenicity at Three Probe Inclination Angles: 0°, −5°, and +5°

Regarding Table 1, the echogenicity on transversal scan at a probe inclination angle of 0° was 176.4 ± 31.32.

Moreover, Table 2 reports the echogenicity on longitudinal scan, showing that, at a probe inclination angle of 0°, the echogenicity was 176.5 ± 30.79.

#### 3.1.2. Echogenicity: Comparison Between Three Probe Inclination Angles on Both Transversal and Longitudinal Scans: 0°, −5°, and +5°

**Figure 3:**
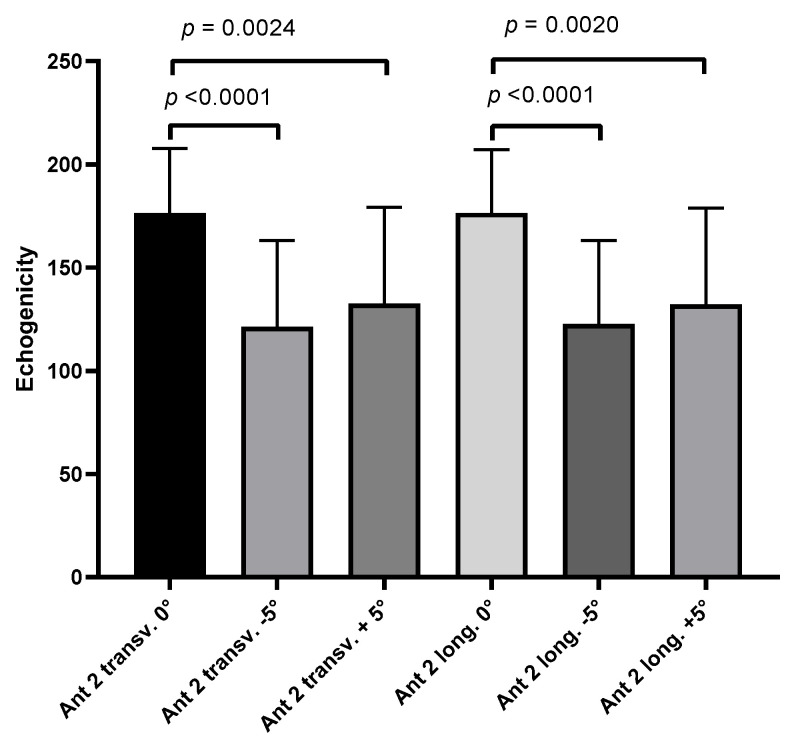
Echogenicity of deep fascia of thigh on transversal and longitudinal scans at three different probe inclination angles (0°, −5°, and +5°).

**Figure 4:**
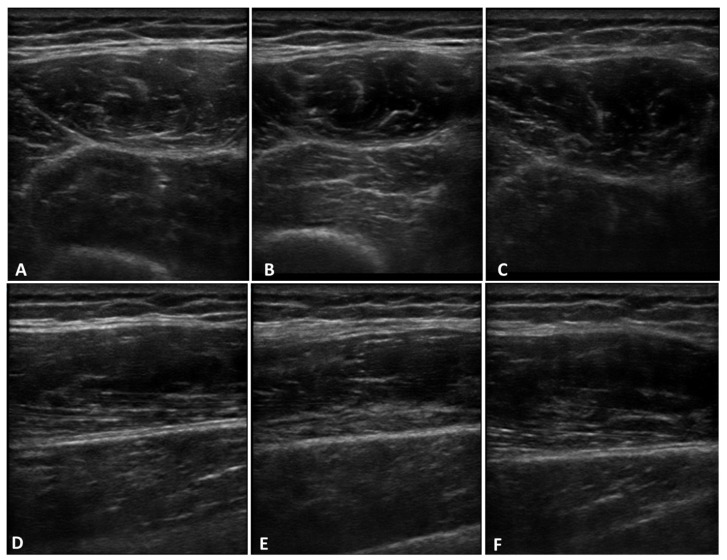
US images of deep fascia of thigh on transversal (**A**–**C**) and longitudinal (**D**–**F**) scans at three different probe inclination angles: 0° (**A**,**D**), −5° (**B**,**E**), and +5°(**C**,**F**).

The comparison between the three probe inclination angles (0°, −5°, and +5°) on both transversal and longitudinal scans showed a statistically significant difference in the echogenicity (Figure 3 and Figure 4).

### 3.2. Ultrasound Measurements of Deep Fascia of Thigh: Thickness

#### 3.2.1. Thickness at Three Probe Inclination Angles: 0°, −5°, and +5°

As show in Table 3, the thickness on transversal scan at a probe inclination angle of 0° was 1.152 ± 0.272 mm.

Moreover, Table 4 reports the thickness on longitudinal scan, showing that, at a probe inclination angle of 0°, the thickness was 1.096 ± 0.267 mm.

#### 3.2.2. Thickness: Comparison Between Three Probe Inclination Angles on Both Transversal and Longitudinal Scans: 0°, −5°, and +5°

**Figure 5:**
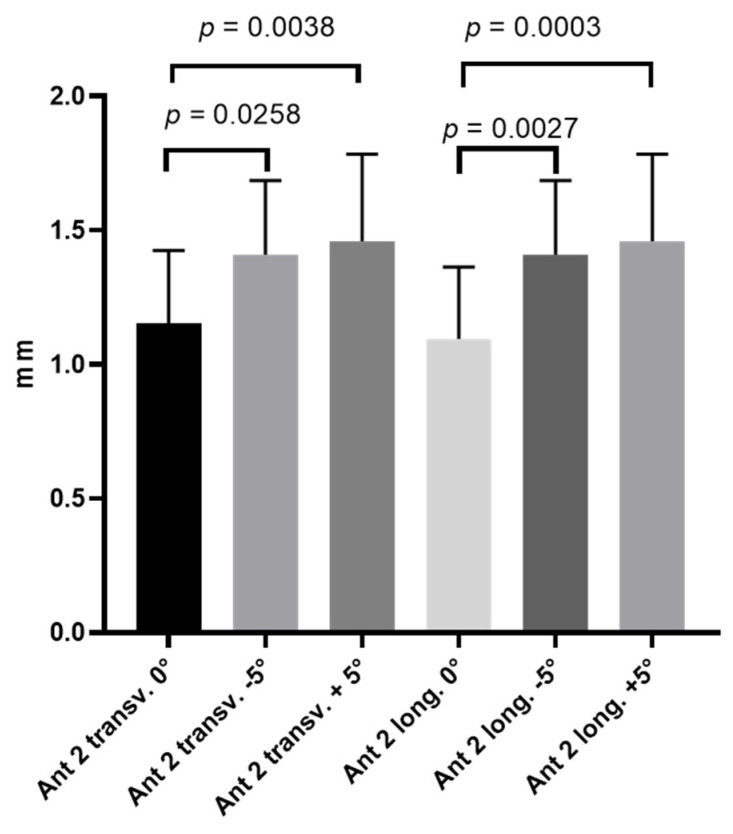
US thickness of deep fascia of thigh on transversal and longitudinal scans at three different probe inclination angles (0°, −5°, and +5°).

The comparison between the three probe inclination angles (0°, −5°, and +5°) on both transversal and longitudinal scans showed a statistically significant difference in the US thickness (Figure 4 and Figure 5).

### 3.3. Intra-Rater Reliability

The intra-rater reliability was evaluated and found to be consistently good to excellent across various conditions (probe inclination angles and axis orientations). Specifically, when the probe was positioned at 0°, the ICC_3,k_ was 0.90 for the longitudinal axis and 0.92 for the transversal axis, demonstrating excellent reliability. In the same way, when the inclination angle was adjusted to −5°, the ICC_3,k_ remained high, with values of 0.91 for the longitudinal axis and 0.92 for the transversal axis, confirming the stability of the measurements despite the slight angular variation. Even with a +5° inclination, the ICC_3,k_ showed minimal variation, maintaining string reliability at 0.90 for the longitudinal axis and 0.91 for the transversal axis. These findings suggested that the measurements were robust and reproducible across different probe positions (Table 5).

## 4. Discussion

Based on our current knowledge, the present study provides, for the first time in the literature, a comprehensive analysis of the impact of probe angulation on echogenicity and thickness measurements in the context of deep fascia, addressing key methodological challenges in musculoskeletal US examination. By quantifying the effects of spatial anisotropy, these findings offer insights into optimizing probe orientation to enhance imaging accuracy and clinical reliability.

The present study’s findings confirm that even minimal deviations from a perpendicular US beam can alter the echogenicity of the deep fascia (Table 1, Table 2, Table 3 and Table 4). Both transversal and longitudinal scans showed marked sensitivity to small probe angulations, highlighting the strong anisotropic behavior of the deep fascia. This is consistent with previous studies demonstrating that collagen-rich tissue exhibits directional variations in echogenicity, attributable to the preferential alignment of fibrillar components [13,14,15]. Furthermore, longitudinal scan at 0° produced no significant difference in echogenicity compared to transversal scan at 0°, suggesting that, when held perpendicular to the fascial plane, both probe orientations provide comparable grayscale intensity. These results emphasize the necessity of strict probe alignment to ensure accurate echogenicity assessments and avoid misinterpretations of tissue integrity.

In the same way, probe orientation had a significant impact on fascial thickness measurements. Deviations from perpendicularity, even as small as ±5°, led to measurable alterations in the estimated thickness. This effect can be attributed to the introduction of beam-width artifacts and reverberation phenomena [13,14,15], which can contribute to overestimation or underestimation of fascial thickness due to increased scatter and secondary reflections. Given that the deep fascia serves as a crucial structural and functional component in biomechanics, erroneous thickness assessments due to anisotropic distortions could have implications in clinical diagnostics and therapeutic monitoring.

The observed data support the conclusion that the most reliable imaging results are obtained when the probe is positioned perpendicularly to the fascial layer. Both echogenicity and thickness measurements exhibited the least variability at 0° probe inclination, in particular at the longitudinal orientation. This finding aligns with previous research on musculoskeletal US artifacts like the tendon, where, in one study, anisotropic effects were minimized by maintaining an optimal probe angulation [13,14,15,16,17,18]. The clinical implications of these findings underscore the necessity of standardizing probe orientation in diagnostic protocols [10,19,20,21]. Given the pronounced impact of minor angulation deviations, systematic training in probe handling and real-time feedback mechanisms, such as adaptive beamforming techniques, may further enhance imaging precision [19,20,21,22,23,24,25,26,27]. In addition, spatial compounding and tissue harmonic imaging have shown potential in mitigating anisotropic distortions [22,23,24,25,26,27,28,29,30], offering avenues for further research in optimizing musculoskeletal US methodologies.

The implications of these findings extend beyond the technical aspects of US examination, directly impacting clinical decision-making in fascial diagnostics. In conditions such as fascial thickening associated with chronic pain syndromes or fibrotic changes following trauma or surgery, the accurate quantification of fascial dimensions is crucial for both diagnostic and therapeutic interventions. Small deviations in probe angulation, if unaccounted for, may lead to diagnostic inconsistencies, misclassification of pathological findings, or ineffective monitoring of treatment outcomes.

Form a broader perspective, these findings support the need for enhanced ultrasonographer training and the development of standardized canning protocols that account for probe positioning and angle-dependent variations.

Furthermore, while this study was conducted under controlled experimental conditions, real-world clinical settings introduce additional challenges, including patients’ movement and variations in scanning environments. Future investigations should aim to validate these findings in dynamic settings, such as during functional movements or weight-bearing conditions, to better understand the clinical relevance of anisotropic distortions in everyday practice.

### Study Limitations

Despite its strengths, this study has certain limitations that should be acknowledged. First, tissue compliance, hydration status, local variations in mechanical tension, age, and pathological conditions could further influence the anisotropic properties of deep fascia, introducing additional variability in US assessments. Future studies should consider incorporating elastography techniques to assess the relationship between fascial stiffness and its anisotropic behavior under different mechanical loads. Another limitation is the focus on the deep fascia of thigh. While the study’s findings likely extend to other topographical regions, applying them as such may result in different degrees of anisotropic distortion across body sites. Comparative studies involving multiple topographical regions could provide a more comprehensive understanding of how anisotropy affects fascial imaging across different anatomical contexts.

## 5. Conclusions

In conclusion, this study systematically demonstrated that probe angulation has a critical impact on deep fascia ultrasonography. First, even minimal deviations from perpendicular probe orientation significantly altered the echogenicity of the deep fascia, confirming its marked anisotropic behavior. Second, fascial thickness measurements were found to be highly sensitive to probe inclination, with non-perpendicular positions leading to substantial measurement variability. Third, the results identified that maintaining a strict perpendicular orientation of the US beam relative to the deep fascia minimizes anisotropic distortions and ensures greater accuracy and reproducibility in US examination. By fulfilling the three primary objectives of this research, this study provides objective criteria for optimizing probe handling during deep fascia US examination, thus contributing to improved diagnostic accuracy and reliability. The inherently protean character of anisotropy underscores the necessity of methodological precision to faithfully capture the true structural features of deep fascia.

## Figures and Tables

**Table 1:** Echogenicity of deep fascia of thigh at the Ant 2 level at different probe inclination angles on transversal scan.

Descriptive Statistics	Ant 2 (Trans. 0°)	Ant 2 (Trans. −5°)	Ant 2 (Trans. +5°)
Number of values	25	25	25
Minimum	123.1	39.98	42.08
Maximum	244.6	189.1	253.7
Mean	176.4	121.3	132.7
Std. deviation	31.32	41.80	46.61

**Table 2:** Echogenicity of deep fascia of thigh at the Ant 2 level at different probe inclination angles on longitudinal scan.

Descriptive Statistics	Ant 2 (Long. 0°)	Ant 2 (Long. −5°)	Ant 2 (Long. +5°)
Number of values	25	25	25
Minimum	123.1	39.98	40.08
Maximum	244.6	189.1	253.7
Mean	176.5	122.6	132.2
Std. deviation	30.79	40.61	46.69

**Table 3:** Thickness (mm) of deep fascia of thigh at the Ant 2 level at different probe inclination angles on transversal scan.

Descriptive Statistics	Ant 2 (Trans. 0°)	Ant 2 (Trans. −5°)	Ant 2 (Trans. +5°)
Number of values	25	25	25
Minimum	0.525	0.957	0.863
Maximum	1.536	1.980	2.144
Mean	1.152	1.410	1.459
Std. deviation	0.272	0.275	0.324

**Table 4:** Thickness (mm) of deep fascia of thigh at the Ant 2 level at different probe inclination angles on longitudinal scan.

Descriptive Statistics	Ant 2 (Long. 0°)	Ant 2 (Long. −5°)	Ant 2 (Long. +5°)
Number of values	25	25	25
Minimum	0.525	0.957	0.863
Maximum	1.536	1.980	2.144
Mean	1.096	1.410	1.459
Std. deviation	0.267	0.275	0.324

**Table 5:** Intra-rater reliability of the ultrasound deep fascia thickness measurements at different scans and probe inclination angles.

Type of Axis and Probe Inclination Angle	ICC
Ant 2 (long.; 0°)	0.90 (0.89–0.94)
Ant 2 (transv.; 0°)	0.92 (0.88–0.96)
Ant 2 (long.; −5°)	0.91 (0.88–0.94)
Ant 2 (transv.; −5°)	0.92 (0.88–0.96)
Ant 2 (long.; +5°)	0.90 (0.89–0.94)
Ant 2 (transv.; +5°)	0.91 (0.89–0.96)

## Data Availability

The data presented in this study are available upon request from the corresponding authors. The data are not publicly available due to privacy reasons.

## Abbreviations

The following abbreviations are used in this manuscript:

US	Ultrasound
MRI	Magnetic resonance imaging
STROBE	Strengthening the Reporting of Observational Studies in Epidemiology
CAD	Computer-aided design
